# Type III Dens Invaginatus with an Associated Cyst: A Case Report and Literature Review

**DOI:** 10.5005/jp-journals-10005-1098

**Published:** 2010-04-15

**Authors:** SM Meghana, P Thejokrishna

**Affiliations:** 1 Lecturer, Department of Oral Pathology, Terna Dental College and Hospital, Navi Mumbai, Maharashtra, India; 2 Lecturer, Department of Pedodontics, Terna Dental College and Hospital, Navi Mumbai, Maharashtra, India

**Keywords:** Dens invaginatus, Pulp vitality, Periapical cyst.

## Abstract

Dens invaginatus (dens in dente) is a rare malformation with a widely varied morphology. An unusual presentation of a type III dens invaginatus affecting a conical shaped permanent lateral incisor in an 8-year-old female patient is reported. The presence of a pulp stone and a periapical radiolucency further added onto the complexity of the case. The etiology, pathophysiology, association with other dental anomalies as well as the challenges in management of this anomaly are discussed. An extensive literature review is also presented.

## INTRODUCTION

Dens invaginatus is a rare developmental anomaly which presents with a widely varied morphology. It has also been referred to as dens in dente, dialated composite odontome, gestant odontome, etc. According to some investigators, the term ‘dens in dente’, originally applied to a severe invagination that gives the appearance of a tooth within a tooth, is a misnomer although it has continued in usage. ^1^ Hallet introduced the term “dens invaginatus” to clarify the point that in invagination, enamel is located centrally and the dentin peripherally. Since then, it has been the preferred term.^2,3^

It can affect both primary and permanent teeth and its prevalence is reported to be 1.7 to 10%. Males are more affected by a ratio of 3:1. All the published studies found that maxillary lateral incisors were the most commonly affected teeth, followed in descending order by permanent central incisors, canines and molars.^4,5^ Other dental anomalies observed in association with dental invagination, including taurodontism, microdontia, supernumerary teeth, germination and dentinogenesis imperfecta.^4,6,7^

**Fig. 1:**
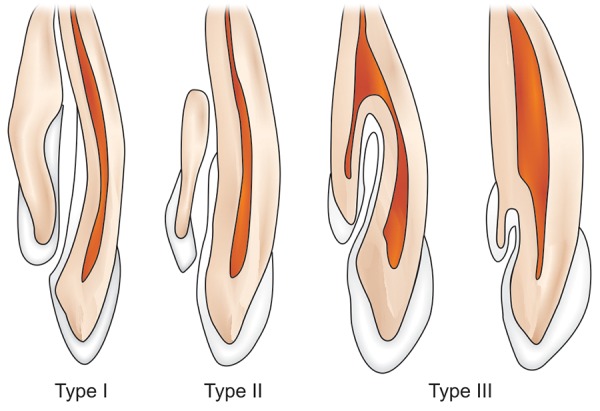
Types of dens invaginatus (Oehler’s classification)^3^

Two types of invaginations are recognized: Coronal and radicular.^5^ The first suggested classification can be credited to Hallet (1953), but the most widely applied classification was suggested by Oehlers who described this condition thoroughly in three classic articles which published from 1957 to 1958 (Fig. 1).^2,4^ The latest classification in this regard was proposed by Schulze and Brand (1972), which is more detailed and illustrating a total of 12 different categories of this anomaly.^8^

## CASE REPORT

**Fig. 2:**
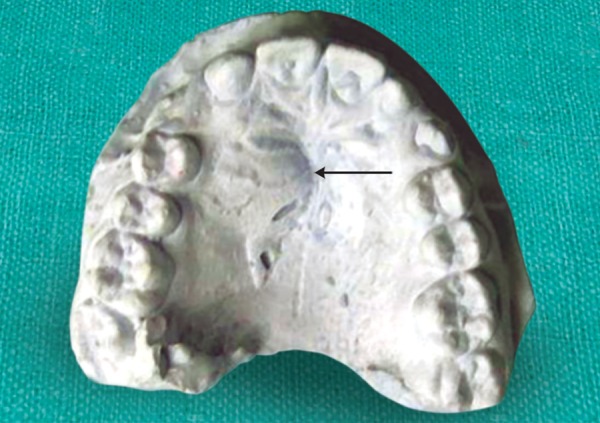
Maxillary cast showing a large conical tooth in relation to 22 associated with a palatal swelling (arrow)

**Fig. 3:**
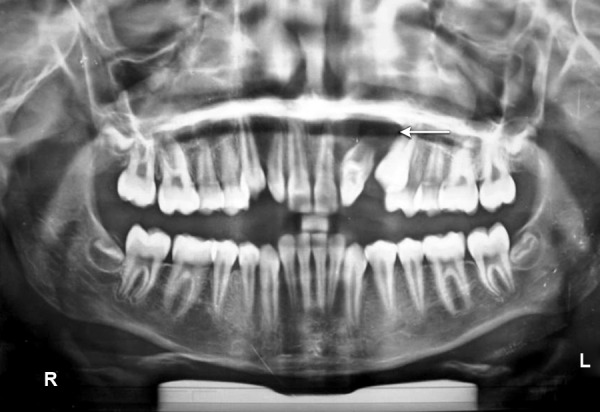
Panoramic radiograph showing a dens invaginatus in relation to 22 associated with a periapical radiolucency surrounded by a sclerotic border (arrow)

**Fig. 4:**
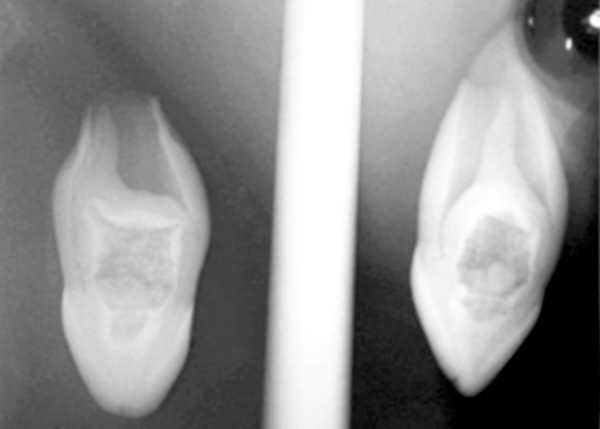
Periapical radiograph (labial and mesial views) of 22 showing a type III dens invaginatus, a main canal, pseudo canal, invagination and a large pulp stone

**Fig. 5:**
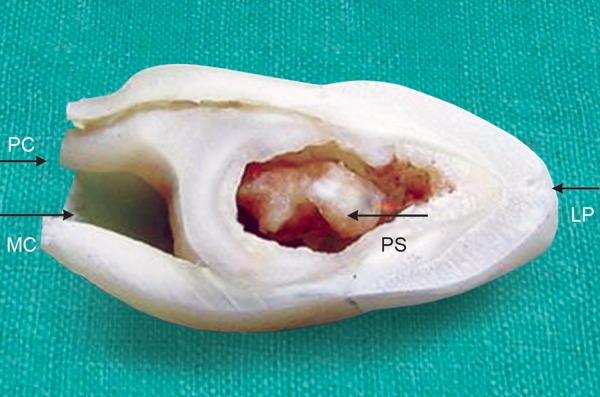
Longitudinal section of the tooth showing a deep invagination extending from the lingual pit (LP). Also seen is the main canal (MC), pseudo canal (PC) and a large pulp stone (PS)

A 12-year-old female patient reported with a complaint of swelling in the left palatal region since 3 months (Fig. 2). The swelling was asymptomatic in nature. The patient’s medical history and extraoral examination was unremarkable. Intra-orally, a swelling firm in consistency was seen on the left side of the hard palate extending from 22 to 24, measuring about 1 cm in diameter. The teeth were free of caries and discoloration. However, 22 did not respond to thermal pulp testing. It was noted that 12 was microdontic and peg-shaped. On the other hand, 21 appeared macrodontic and dialated. Panoramic radiograph revealed the presence of a periapical radiolucency surrounded by a sclerotic border in relation to 22. The clinically missing 23 was seen ready to erupt and 22 showed the presence of a wide pulp chamber which contained a large irregular radiopacity which corresponded to a pulp stone (Fig. 3). There appeared to be a radiopaque invagination from a lingual pit towards the root apex crossing the cement-enamel junction. This invagination was approximately circular with a central core of radiolucency, which was consistent with the diagnosis of a dens invaginatus type III, containing a pulp stone (Figs 4 and 5). It was decided to extract the tooth, since the presence of a huge pulp chamber, large pulp stone and the periapical radiolucency will complicate the endodontic treatment. The enucleated cyst upon histopathological examination was diagnosed as a periapical cyst.

## DISCUSSION

Development of an invaginated tooth has been explained based on a number of hypotheses. The earliest hypothesis attributed the malformation to the incomplete fusion of two tooth germs or to the attempted division of a single tooth germ (Bruszt et al). Also suggested, there was an abnormal proliferation of enamel organ invading the dental pulp (Oehlers, 1957). However, some authors also considered the invagination to be a deep infolding of the apical foramen during odontogenesis, resulting in some cases, a second apical foramen (Schulze C, 1970). Other proposed theories include altered tissue pressure during odontogenesis, trauma, infection and localized discrepancies in cellular hyperpla-sia. A recent hypothesis proposes dental invagination as a consequence of the degeneration of the dental lamina which can lead to fusion, gemination or agenesia. ^4^ This theory is supported by the fact that invagination is more common in maxillary lateral incisors and premolars, along with the other anomalies.

The genetic and syndromic association with dens in-vaginatus has always been debated upon without much clinical evidence available so far. Mann et al considered the anomalies found in a patient to be a variant of Ekman-Westborg-Julin syndrome, where multiple anomalies in dental morphology were found, including macrodontia, mul-tituberculism, central cusps and pulp invaginations. Polaka and Acs reported a case where the patient was diagnosed with a chromosomal abnormality in which the long arm of chromosome 7 was found deleted at a highly specific locus (7q32). The patient, in addition to having craniofacial abnormalities and developmental delay, presented with numerous clinically and radiographically evident dental anomalies, including dens invaginatus.^2^

The most significant clinical concern of dens invagi-natus is the risk of developing pulpal pathology. The in-vagination commonly communicates with the oral cavity, allowing the entry of irritants and microorganisms directly into the pulpal tissue. Although enamel lines the coronal defect, it is frequently thin, often of poor quality and even missing in some areas. The pit is often difficult to keep clean, and consequently, it offers conditions favorable for development of caries, usually leading to the necrosis of the adjacent pulpal and periapical tissues. Also, sometimes fine canals extend between the invagination and the pulp chamber, resulting in pulpal and periapical pathology even in the absence of dental caries.^9^ If the invagination extends from the crown to the periodontal tissue and does not communicate with the root canal system, the pulp can remain vital. Pulpal involvement can occur at a young age, when the root is immature and not completely formed. In these cases, the difficulty is to facilitate the apical closure before obturation. Other reported sequelae of undiagnosed and untreated invaginated teeth including eruption delay, cysts and internal resorption.^2,4^

Depending on the severity and extent of the malformation, the treatment may vary from a prophylactic fissure sealing to root canal or extraction. Until the 1970s, extraction of teeth with severe invagination was the treatment of choice and it still is, especially when the abnormal crown morphology presents esthetic or functional problems, as proposed by Rotstein et al (1987).^3^ Nonsurgical root canal treatment should be attempted first. Irrespective of the size of the periapical lesion, surgical intervention must be the second option and is only indicated when nonsurgical root canal treatment has failed or the anatomic variations of the canals do not allow access for the biomechanical preparation of the canals. Periapical surgery is indicated in cases of unsuccessful apexification in immature teeth with dens invaginatus and nonvital pulp. ^3,10^

## CONCLUSION

Dens invaginatus can often be a concealed finding. Therefore, we suggest radiographic examination of all teeth that present with shape anomalies and hypoplasia especially maxillary anteriors. Further, upon radiographic finding of dens invaginatus, the apical periodontium must be carefully examined. The complexity of the root canal system, open apex and pulp stones in dens invaginatus presents a challenge to endodontic treatment. Since there is a high incidence of pulp infection and degeneration associated with this anomaly, early diagnosis to prevent pulp necrosis and pulpal inflammation becomes critical.^11^ Eventually, the loss of the tooth and the potential complications following the loss can be prevented. The most significant issue remains the correct diagnosis and subsequent proper management on the basis of sound biologic and clinical principles.

## References

[1] Rajendran R., Sivapathasundharam S. (2005). Developmental disturbances of oral and paraoral structures. In: Shafer’s Textbook of Oral Pathology..

[2]  Mupparapu M, Singer SR (2006). A review of dens invaginatus (dens in dente) in permanent and primary teeth: A case report in a microdontic maxillary lateral incisor.. Quintessence Int.

[3] Kristoffersen Ø,  Nag OH, Fristad I (2008). Dens invaginatus and treatment options based on a classification system: Report of a type II invagination.. Int Endod J.

[4]  Galindo-Moreno PA, Parra-Vazquez MJ, Sanchez-Fernandez E, Avila-Ortiz GA (2003). Maxillary cyst associated with an invaginated tooth: A case report and literature review.. Quintessence Int.

[5] Neville B., Damm DD., Allen CM (2005). Abnormalities of teeth. In: Textbook of Oral and Maxillofacial Pathology..

[6]  Ireland EJ, Black JP, Scures CC (1987). Short roots, taurodontia and multiple dens invaginatus.. J Pedod.

[7]  Morfis AS (1993). Chemical analysis of a dens invaginatus by SEM microanalyses.. J Clin Pediatr Dent.

[8]  Chaniotis AM, Tzanetakis GN, Kontakiotis EG, Tosios KI (2008). Combined endodontic and surgical management of a mandibular lateral incisor with a rare type of dens invaginatus.. J Endod.

[9] White SC., Pharoah MJ. (2004). Dental anomalies, oral radiology. In: Principles and interpretation..

[10]  Fregnani ER, Spinola LF, Sonego JR, Bueno CE, De Martin AS (2008). Complex endodontic treatment of an immature type III dens invaginatus: A case report.. Int Endod J.

[11]  Zengin ZA, Sumer PA, Celenk P (2009). Double dens invaginatus: Report of three cases.. Eur J Dent.

